# A community-based, sport-led programme to increase physical activity in an area of deprivation: a qualitative case study

**DOI:** 10.1186/s12889-020-08661-1

**Published:** 2020-06-29

**Authors:** Aphra Garner-Purkis, Samah Alageel, Caroline Burgess, Martin Gulliford

**Affiliations:** 1 King’s College London, School of Population Health and Environmental Sciences, London, UK; 2 King Saud University, College of Applied Medical Sciences, Community Health Sciences Department, Riyadh, Saudi Arabia

**Keywords:** Exercise referral scheme, Physical activity, Health promotion, Exercise, Sedentary lifestyle, Social prescribing, Primary care, Social enterprise

## Abstract

**Background:**

Engaging in physical activity is essential for maintaining mental and physical health but a high proportion of adults are inactive, especially in areas of socioeconomic deprivation. We evaluated a novel exercise referral scheme funded by Sport England and run by a social enterprise in an area of socioeconomic deprivation in inner London. This study aimed to examine the experiences of participants and staff and to identify barriers and facilitators of implementation and participation in this and potentially similar projects.

**Methods:**

Thirty-five semi-structured interviews with project participants (*N* = 25) and staff members involved with the project (*N* = 10) were conducted based at one centre in London in 2017/2018. The interview schedule was informed by the Theoretical Domains Framework. Data was analysed using the Framework method and NVivo software.

**Results:**

Three themes emerged from the data: *‘Not like your regular gym’*, *Individual journeys* and *Practical aspects of the scheme*. Study participants regarded the environment of the project centre as friendly and sociable. The project differed from a commercial gym by offering free or subsidised membership and the participation of people of all sizes and abilities. Classes were provided free of charge and this, together with mentor support, facilitated participation and continuation in the project. Participants reported changes not only in their physical activity level, but also in their physical and mental health. Additionally, their families’ lifestyle changes were reported. Difficulties of accessing the project included lack of awareness of the project and lack of engagement from key referring groups.

**Conclusions:**

Providing free or subsidised classes incorporating individualised assessment, follow-up and support appeared to facilitate engagement in physical activity among socioeconomically deprived populations. The supportive social context of the centre was a major facilitator. Differing levels of abilities and health status among participants call for special attention. Increasing community and referrer awareness of available exercise referral schemes and enhancing communication between sources of referrals and project staff may help to address access issues.

## Introduction

Physical activity is associated with reduced risks of obesity, cardiovascular diseases, type 2 diabetes, cancer and mortality [1]. In the United Kingdom, national physical activity guidelines recommend that adults should be active every day with at least 150 min of moderate or vigorous activity per week [2]. A target of 5 days per week with at least 30 min of activity is widely recommended [2]. However, according to the World Health Organisation, one in four adults worldwide are insufficiently physically active [1]. In England, 25.2% of adults were *‘*physically inactive’ in 2017 with less than 30 min of physical activity per week [3]. Women [4–6], older people [5, 7], non-white individuals [6, 7] and those living in socioeconomically deprived neighbourhoods [8] are more likely to be inactive.

Exercise referral schemes (ERS) represent a form of social prescribing that have been first set up around 1990 in the United Kingdom and is growing rapidly since then [9]. ERS have been used in the context of health care services in attempts to increase physical activity in key target groups such as people with an elevated body mass index or people with specific conditions, for example depression or stroke [10]. Previous research on ERS found differences based on sociodemographic factors between individuals who are more or less likely to be referred, take up the referral, adhere and complete the scheme and achieve changes as a result [5, 11, 12]. For instance, older adults were more likely to take up the referrals and complete ERS compared to younger age groups [5, 12]. Other differences were observed in relation to existing health conditions, where people with pulmonary diseases were less likely to complete ERS than people with cardiovascular diseases [12]. Favourable aspect found in ERS research findings are the presence and importance of social support for participants [13, 14]. Other factors were reported as barriers to whether participants take up the referral and complete ERS, including the timing of sessions, cost and location, awareness of the project, competing demands and intimidating/ unappealing/ uninviting gym environments [14, 15]. Facilitators may include: internal motivation, support from providers, financial incentives, supportive environments and modified eating habits [14, 16]. Most of previous research has been through randomised controlled trials or local evaluations. There is a lack of qualitative research into ERS that could help identify factors that may have affected engagement and outcomes of such schemes. Previous evidence has mainly focused on specific population experiences (e.g. people with disabilities), and only from the participants’ perspective [14]. Combining participants’ perspectives with the scheme providers, including referrers, to identify a range of factors associated with referring participants, scheme implementation, attendance and adherence to the scheme. This could inform future developments of the same project or for the project’s use in another setting or population.

This study addresses the need to develop, implement and evaluate population-level interventions to increase physical activity with a focus on increasing engagement in exercise among individuals that are presently physically inactive. The present study reports a novel community-based ERS that was open to physically inactive residents in five socioeconomically deprived electoral wards in an inner London borough. The scheme, known as *Active Lifestyles for All (ALFA),* was one of 16 projects funded through Sport England’s *Get Healthy Get Active* scheme. This qualitative study aimed to explore the experiences of participants and staff involved in an ERS and to identify barriers and facilitators to the participation and the implementation of such projects.

## Methods

### ALFA project description

The ALFA project was a three-year community-led intervention in one London Borough that was managed by a social enterprise with expertise in sport education. The ALFA project was targeted at residents in five socioeconomically deprived electoral wards. All activities took place at the social enterprise’s new ‘healthy lifestyle centre’ in a regenerated playing field in the neighbourhood, which included exercise facilities, a café and space for social interaction. The location was in a quieter residential area a distance from main roads. It aimed to encourage physically inactive people aged 14 years and above to become and stay active. Potential participants were screened for eligibility using the Single Item Questionnaire for Physical Activity and were identified as inactive (i.e. completed 30 min or less of moderate activity a week). The ALFA project recruited a total of 1416 participants (including 1216 women and 200 men) between September 2015 and June 2018. The largest number of participants (618, 44%) were self-referred and the remaining participants were referred from the National Health Services including GPs, pharmacies and mental health services, public health services in the Borough, and community organisations for older people and people who are overweight and obese. There were 212 (15%) aged 65 years and older and 15 (1%) aged less than 18 years. The distribution of participants by ethnic group generally represented the demographic make-up of the local area, with approximately equal numbers of ‘white’ (476, 34%) and ‘black Caribbean and black African’ (485, 34%) ethnicity and smaller representations of Asian, mixed, other and not stated ethnic groups. Deprivation was evaluated using the Indices of Multiple deprivation for 2015, based on participants’ residential Lower Super Output Area, there were 486 (34%) in the lowest 5th of deprivation and 496 (35%) in the second lowest 5th of deprivation. Sport activity was recorded at baseline for 1265 (89%) participants using the *International Physical Activity Questionnaire (IPAQ*) questionnaire.

The project involved trained health mentors working with physically inactive residents to develop individually tailored exercise and health plans for becoming active through a choice of a range of sport activities. Mentors met with their project participants initially to discuss goals and take baseline physical activity assessments*.* Sports activities varied to suit a range of people of different physical abilities including yoga, fitness trampoline classes and Zumba with the option of a seated version for those less able to stand. The mentors’ role included supporting participants to engage in their selected sport activities for at least 30 min (i.e. one session) per week for 13 weeks free of charge. Subsequently, sessions were offered at a reduced rate. The project had no explicit theoretical framework and the fidelity of implementation was not explicitly addressed. There were 1110 (88%) who reported sport participation on no days per week; 125 (10%) participated on 1 day per week; and 30 (2%) participated on two or more days per week. Sport activity was recorded at 3 months for 1013 (72%) participants. There were 661 (65%) who reported sport participation on no days per week; 267 (26%) participated on 1 day per week; and 85 (8%) participated on two or more days per week.

### Evaluation design

This study employed qualitative methodology, applying framework analysis to data collected from semi-structured interviews with stakeholders involved in the project. The study was approved by Kings College London Ethics Committee (Ref no. LRS14-15-1659).

#### Participant recruitment

There were two distinct groups of respondents namely: project participants and staff. Project participants were individuals that had been recruited into the ALFA project and had taken part or were currently taking part in their 13 activity sessions. They were recruited via the project manager who acted as a gatekeeper informing people of the study for the research team to then contact. The staff group were further distinguished into referrers, mentors and other staff. Referrers worked for external organisations that referred individuals to the ALFA project and included GP’s and community workers. Mentors on the ALFA project were staff members who were allocated to supervise a group of project participants. Other staff respondents in the scheme included those with management and consultation roles for the ALFA project. The project participants and staff were sampled for the interviews by the ALFA project manager using convenience and subsequently snowball sampling. Recruitment was planned to continue until no new themes arose and data saturation has been reached.

#### Interview schedule

The development of the interview schedule (Additional file 1) drew on the Theoretical Domains Framework (TDF) [16]. The TDF is an integrated framework that aims to provide a comprehensive, theory-informed approach to identifying influences on behaviour, the TDF has been used in a range of studies [17]. In the present study, the TDF was employed with the aim of enabling the identification of factors that participants may not otherwise report [18]. For this study the most recent framework was used which included 14 domains, covering a broad range of individual and organizational theories [19]. By using this framework, it limited the possibility of missing important factors affecting the participation in the project. The topic guide was discussed and edited by three experts in the field with experience in using the TDF in qualitative research.

#### Data collection

Interviews were conducted face-to-face at either the ALFA project centre, or at the staff member’s workplace. Interviews were conducted face-to-face, with the exception of two interviews conducted over the phone. Information sheets were sent to individuals prior to the interview and written consent was taken at the beginning of each interview. Verbal consent was taken for the telephone interviews. Interviews were conducted by one researcher (AGP) between October 2017 and February 2018. The interviews lasted from 10 to 55 min.

#### Data analysis

The interviews were audio-recorded and transcribed verbatim. The transcripts from the interviews were coded and managed using NVivo software [20] and the coded data were managed and classified using Framework Analysis [21]. The Framework Analysis process follows seven steps: transcription, familiarization, coding, developing and then applying an analytical framework, charting data and data interpretation [21]. The interview transcripts were coded initially and refined by the first author (AGP) and checked by another author (SA) for consistency and a third author intended to resolve any remaining disagreement. Face-to-face meetings were conducted to discuss codes, and amendments were made where appropriate until full agreement was reached. The coded data were then examined in relation to the TDF and tables were produced for the development of the analytical framework. Data interpretation and the creation of themes and sub-themes was initially completed by the first author (AGP) and then checked, discussed and refined with the research team.

## Results

Thirty-five individuals were interviewed. The majority were project participants (*n* = 25), the staff group included mentors (*n* = 4), referrers (*n* = 3) and managers and consultants for the project (n = 3). Project participant demographics are summarised in Table 1. Twenty-four of the twenty-five project participants in our study were women. This proportion was comparable to the overall proportions in the project, where 89% were women. This made any gender differences of the project difficult to reflect upon. Staff characteristics are summarised in Table 2. Seven of the ten staff interviewed were women. Most staff were involved from the beginning of the project while only one had become involved with the project within the past 2 months. Three themes emerged from the data: ‘*Not like your regular gym’, Individual journeys* and *Practical aspects of the scheme*.

**Table 1:** Project participant characteristics

Characteristic	Freq. (%)
**Sex**	
Men	1 (4)
Women	24 (96)
**Age**	
25–34	1 (4)
35–44	3 (12)
45–54	9 (36)
55–64	9 (36)
65–74	1 (4)
75–84	0
85	1 (4)
Not stated	1 (4)
**Ethnic group**	
White	13 (52)
Mixed/multiple ethnic groups	2 (8)
Asian/Asian British	1 (4)
Black African	1 (4)
Black Caribbean	6 (24)
Other ethnic group	2 (8)
**Employment status**	
Employed	16 (64)
Unemployed	1 (4)
Carer	2 (8)
Retired	5 (20)
Employed/retired	1 (4)
**Interview type**	
In person	23 (92)
Phone	2 (8)

**Table 2:** Project Staff characteristics

Characteristic	Freq. (%)
**Sex**	
Men	3 (30)
Women	7 (70)
**Role in project**	
Mentors	4 (40)
Referrers	3 (30)
Manager/consultant	3 (30)
**Period of involvement**	
Beginning of the project	7 (70)
2 months – 1 year after project start	2 (20)
Recent involvement (past 2 months)	1 (10)

### Not like your ‘regular’ gym

This theme (Table 3) reflects on how the scheme differed from ‘regular’ gym experiences including social aspects of the project, the effect of having one-to-one mentor support and other specific ways it differed from a gym in clientele and setup.

**Table 3:** Themes, sub-themes and illustrative quotes

Theme	Sub-theme	Content	Evidence
**‘Not like your regular gym’**	**Social aspects**	Healthy Lifestyle Centre as friendly, welcoming, safe and sociable.	*‘And the kids are fine sat there, aren’t they, you know? You don’t mind them sitting down on the sofa out there*.’
		Peers as role models.	*‘It was really great for me, because yes, having these role models of like people who keeping coming, keeping coming every week.’*
	**Role of mentors**	Mentors dependable but encouraging self-sufficiency.	*‘[for mentors] it’s a tightrope, you’ve got to find that balance between encouragement and not pressurising [clients].’*
		Activities tailored to needs.	‘*different people need different advice and different guidance.’*
	**Differences to a gym**	Affordable.	*‘And you think, well what if I don’t like [private gym membership], I’m just throwing money away.’*
		Non-competitive.	‘*No posing people… don’t feel being watched or looked …. a lot of people don’t like to go to the gym because they feel a bit scared.*’
			‘*there’s other people like me who’ve not done classes, or, you know, are not fit. And so just accept that you know there’s going to be other people in the room that are similar to you*.’
	**Social responsibility**	Duty to funders	*‘I kind of felt well I’ve signed up for this and someone else is paying for this, so I should go. That’s my responsibility to go to it because it’s a good opportunity.’*
**‘Individual’s journey’**	**‘Get that motivation going’**	Deciding to become more healthy through physical activity and sport.	‘…. *obviously [clients] made that sort of step in their mind that they need to get more healthy.’*
			‘*As you get older, you kind of slow down and even though I don’t look that old, I feel old. So it’s important for me to look after myself as I get older.’*
	**‘Different things going on in their lives’**	Project in the context of people’s lives.	*‘when I’d lost the whole structure of my life really……something for me to build on.’*
			‘*But I just find that I get really get into it, but then if my health’s not so great, then obviously I don’t exercise for a few weeks, and then I’m back to square one. I sort of lose the motivation a little bit*.’
	**‘It has changed my life’**	Changing usual activity patterns.	*‘…my son and my daughter – I sort of say to them, you know ‘Get up, let’s go round the block. You know if they can walk somewhere they’re not always going to get a lift from me in the car. ‘Let’s leave the car and we’ll walk there instead.’*
**Practical aspects**	**Access**	Difficulty from HLC location	‘*So that kind of stopped them from getting to this project, which would have been something that, you know, if they’d got the transport sorted out, it would have really ran to its maximum.’*
	**Community awareness**	Need to promote project	‘*I don’t remember anyone going, ‘Oh yes I’ve heard of them.’ No, I don’t think people knew of it.*’
	**Engaging stakeholders**	Promoting referrals	‘*So, if you could have had them [GP’s] on-board a little bit more and a bit more of a closer relationship, that would have been nice.*’

#### Social aspects

The introduction of the ALFA project had created a sense of local community. This was illustrated by how individuals described new personal connections with people locally that they had made by participating in ALFA classes. Some reported feeling less fearful about their local environment. Project participants and staff regarded the environment at the centre as friendly, welcoming, safe and sociable. The addition of the café in the centre was regarded positively, making it easier to interact with staff more informally and providing a social space for individuals to use. Project participants were influential in encouraging and motivating each other to attend and push themselves physically in exercise classes.

*‘ … was really great for me, because yes, having these role models of like people who are keeping coming, keeping coming every week, that I’d see them. And like just seeing how we could all do it, like without feeling bad about ourselves. We could just kind of come and do exercise, it’s really good.*’ [Project Participant, P62].

#### Mentor and project participant relationship

Contrasting relationships between mentors and project participants were reported. For some a real friendship developed whilst others maintained a more formal relationship. Mentors indicated that they wanted to be a dependable figure for participants during their period of participation, while having made them self-sufficient by the end.

*‘it’s a tightrope, you’ve got to find that balance between encouragement and not pressuring them’* [Mentor, 9]*.*

There was a clear message that was promoted across the project of consistent and open communication between the mentor and participant. Mentors in turn kept track of their participant’s attendance and progress. Project participants considered that mentors were supportive, attentive and encouraged them to take more classes in the project. This also motivated participants to seek additional exercise classes elsewhere.

#### Differences to a gym

The project was described as differing from the gym experience in both setup and clientele. Expensive membership and long commitment periods were viewed as negative aspects of gym engagement. The ALFA project was seen as offering the space to exercise in a safe and comfortable environment. The difference in clientele between gyms and the project was clearly an important factor for project participants. Participants perceived the project as not comprising of ‘posing’ people, they did not feel judgement from others and the classes were made up of a mixture of people of all shapes, sizes and abilities.

‘*There’s other people like me who’ve not done classes, or, you know, are not fit. And so just accept that you know there’s going to be other people in the room that are similar to you*.’ [Project Participant, P19].

Although there were positives in having such a mixture of people attending and a wide range of classes, this was not always manageable and it was apparent that stakeholders realised the project’s capacity. Resource limitations discussed were: the restrictions of the project’s catchment area, the challenges associated with participants with co-morbidities and issues with unsuitable referrals resulting in classes with an extreme mix of abilities. For instance, participants with chronic obstructive pulmonary disease reported difficulties to participate regularly and needed one to one assistance. Others participants with depression and irritable bowel syndrome could not consistently attend, but reported health benefits when they attend a session. In addition to this was a lack of staff skills to deal with specific participants’ medical conditions.

‘*Some of them, as I said, have severe health concerns and conditions that myself or I know other mentors have never even heard of so to try and prescribe fitness around that, not really best qualified to do that*.’ [Manager/consultant, O16].

#### Social responsibility

ALFA project participants expressed how the initial period of free classes acted as an incentive, and that is was their ‘duty’ to make the most out of. Some participants felt that missing classes was an opportunity missed that someone else could have benefited from it.

*“I kind of felt well I’ve signed up for this and someone else is paying for this, so I should go. That’s my responsibility to go to it because it’s a good opportunity.” [Project Participant, P20].*

*“And it just felt like I was taking something away from someone who might otherwise have had it.” [Project Participant, P19].*

For others, they felt responsible to attend the classes and complete the project due to their obligation to their mentors to complete the 13 weeks project.

*“There’s that slight more really like thinking, “Oh I must go, you know, because when she [the mentor], when I meet with her, I’m going to say, “No I’ve only attended three.”” And although she goes, “Oh that’s fine,” you know that they’re going to be disappointed, aren’t they?” [Project Participant, P8].*

### The individual’s journey

This theme (Table 3) dealt with factors at an individual level including personal circumstances, the impact on individuals and how ALFA project participants were engaged and retained.

#### Project in the context of people’s lives

Individuals discussed how their own commitments fit with the project. Those that worked full-time expressed more frequent challenges with attending including: being tired, working late and covering colleagues sick leave. Some ALFA project participants explained how the project had given them a structure and focus after their lives had changed due to either redundancy or retirement

‘ *…when I’d lost the whole structure of my life really ……you know that was my hope …A hook something for me to build on.’* [Project Participant, P19].

Family commitments were explored, those that had younger children had mixed experiences. Some found it difficult organising childcare and exercising in the evenings, feeling that their kids had ‘lost their evening’. For others it fit in more easily with their schedule.

‘*It worked out fantastically actually……So I could drop the children off there and then I’d go there every Saturday morning.’* [Project Participant, P64].

It appeared that individual health conditions could be either a barrier or a facilitator. Almost half of the project participants explained how their health condition acted as a motivator to get them into physical activity and onto the project. Staff acknowledged how physical activity could help with some conditions (e.g. strengthening the body or some mental health conditions). But it was perceived as a barrier for some, resulting in lower attendance and an inability to participate in certain activities.

‘*But I just find that I get really get into it, but then if my health’s not so great, then obviously I don’t exercise for a few weeks, and then I’m back to square one. I sort of lose the motivation a little bit*.’ [Project Participant, P64].

#### Getting that motivation going

Many participants stated that they had self-referred to the ALFA project. Staff perceived self-referred participants as motivated and ready to change.

‘…. *obviously made that sort of step in their mind that they need to get more healthy.’* [Manager/consultant, O16].

Staff believed that participants that were referred by their GPs were often less willing to engage and commit to the project than others. This was not reflected in comments from project participants who were referred by their GP’s, they expressed enthusiasm for the project and appeared to have continued exercising since entering the project.

A range of triggers or motivating factors were reported that pushed individuals to get active. Participants mentioned having previously exercised and their desire to get back into it, some acknowledged getting older and so saw the purpose in increasing their physical activity to prevent future problems.

‘*As you get older, you kind of slow down and even though I don’t look that old, I feel old. So it’s important for me to look after myself as I get older’.* [Project Participant, P20].

Participants also stated that their confidence had increased over the course of the project. Staff discussed project participant motivations and believed that people needed personal motivation to commit to the scheme.

‘*It requires a trigger. If not, you’re probably not going to find the motivation, no matter how, no matter how much the people around you are trying to motivate you*.’ [Mentor, M9].

#### ‘It has changed my life’

A change of mindset from when they started attending and around exercise more generally was reported by participants and was regarded positively

‘*I think the benefit is to get people to actually change their lifestyle, change the way they look at exercise, for one*.’ [Project Participant, P27].

Staff reiterated this point, highlighting the importance and necessity for people to have a desire to change for it to actually happen. This included changing thought patterns, habits and routine which could be challenging to influence in other people.

‘*That’s the most difficult part and it’s the most important part, is changing the mindset.*’ [Mentor, M9].

Lifestyle changes included increased walking and additional exercise, diet changes, encouraging others to exercise and beginning to prioritise exercise. Those with kids (mainly younger kids) also mentioned getting their kids involved with physical activity.

*‘…my son and my daughter – I sort of saw to them, you know “Get up, let’s go round the block…..you know if they can walk somewhere they’re not always going to get a lift from me in the car.” Let’s leave the car and we’ll walk there instead’.* [Project Participant P6].

Physical and mental consequences of increased exercise were reported including improved abilities and fitness levels, increased energy, losing weight, feeling happier, being more able to deal with challenges and feeling more relaxed when exercising after a stressful day. The future commitment to physical activity was clearly an important consideration for participants.

*‘I never stop a day, never ever. So that’s all it is. I just think it’s good.’* [Project Participant, P61].

### Practical aspects of the project

This theme (Table 3) is about how the project operated from access, to the awareness of the project in the community and engaging stakeholders (external staff members) in the project.

#### Access

Most people said the ALFA project location was easy for them to get to, but it was acknowledged that it might be difficult if people did not drive as it was not easily accessible by public transport. Lack of transport was discussed as a barrier to involvement by project participants and staff.

*“So that kind of stopped them from getting to this project, which would have been something that, you know, if they’d got the transport sorted out, it would have really ran to its maximum.”* [Referrer, R9].

It was suggested by staff that one modification to improve the project would be to run it in several external locations as opposed to just one centre.

#### Community awareness of the project

Consensus amongst project participants was that the project was not well advertised and that many people in the local area still did not know about the project. Bearing this in mind, individuals did speak of several ways that they were made aware of the project including: a ward assembly, doctor’s surgeries, the local magazine and referrals by doctors. The most common way people knew of the project was through word of mouth, usually through a friend or family member’s recommendation. A few became aware of the project due to chance encounters in the local park where the class was taking place. Staff generally mirrored these views on the issues of the lack of awareness.

*“I don’t remember anyone going, “Oh yes I’ve heard of them.” No, I don’t think people knew of it.”* [Referrer, R1].

#### Engaging stakeholders

Communication streams within the project and with external organisations showed varied experiences. Internal project staff discussed the positives of working with some referrer organisations, for instance, the provision of additional participant information when required. However, some issues were expressed with the GP referring groups; doctors were not as invested in the project as they could have been.

‘*So, if you could have had them [GP’s] on-board a little bit more and a bit more of a closer relationship, that would have been nice.*’ [Manager/Consultant, O16].

Referrers also reported on the lack of feedback about participants progress and regular updates from the project, which led to ceasing referring people onto the project. The initial awareness and knowledge of the project’s essence and its progress also seemed to vary in referrers experiences, this may have been to do with how well it was communicated. Some knew full details at the projects start whilst others had issues, although there was contact information available they felt there was no real person contact to explain the project and how it would work.

## Discussion

This study aimed to evaluate factors that enabled or impeded participating in a community-based physical activity referral project using stakeholder perspectives. The study found that project participants valued the social aspects of project, including the way the project differed from ‘regular’ gyms in terms of cost, clientele and support provided. These factors acted as motivators to commit to the project and participants also felt a responsibility to complete the project because of the financial incentive and so as not to disappoint their mentors. At an individual level, personal circumstances influenced how they were able to fit the classes in their lives. Health conditions of the participants were acknowledged either as a barrier or facilitator by staff and the project participants. Changes as a result of the project included an increase in participants’ confidence to exercise and changes in their overall lifestyle, including their families. At a wider level, access issues were viewed as important by staff and project participants; the location of the project centre had some drawbacks with its residential positioning. Awareness and promotion of the project was regarded as low, and GPs did not seem to engage in the referral process, therefore missing an important group.

The project’s social environment played a major role in facilitating participants’ engagement with physical activity. The project was perceived as a safe and comfortable environment due to it attracting people of all shapes and abilities. Many studies have suggested an association between the gym environment and feeling intimidated and uncomfortable [14, 22]. Gyms were perceived to typically attract fit, slim and young people and thus an unwelcoming environment for an older and inactive population [14]. The participation of like-minded companions in group physical activity facilitated the enjoyment and integration to physical activity schemes [14]. Providing a variety of classes that is suitable for participants’ differing needs and health conditions motivated the continuation in physical activity, as reported in the current study, and previous evidence [14]. This study’s participants (both project participants and staff) have acknowledged the lack of staff skills in dealing with medical conditions that required special care. This could be the result of overambitious referral strategy and lack of communication between the referrers and the mentors. Therefore, such programmes need to be accompanied by skilled trainers with an experience of dealing with different medical conditions.

The method of recruitment may have played a role on the project participants’ experience. Self-referred participants appeared to be more motivated and engaged with the project. Project staff thought that participants who were referred by their GP were less committed and enthusiastic about the project. It has been previously suggested that the impact of health assessment on behaviour change may vary according to the mode of recruitment, where self-referred participants being more responsive and more likely to report health behaviour change [22]. Self-referred participants might be more prepared to act and motivated to change than actively recruited participants. However, it might be unwise for future projects to rely only on self-referrals because then needier groups, with greater barriers to participation, might not be included.

Social support appears to be an important facilitator in engaging and motivating participants in the project. This came in the form of participants’ relationship with their mentors and fellow participants. The sense of care and continued follow-up was an essential factor to participant engagement, as reported previously [14]. Participants relationship with their mentors created a sense of responsibility and loyalty to continue with the project. Although this factor facilitated the continuation of the project, the impact on the long-term participation in physical activity is unclear. The use of technology-based interventions to facilitate behaviour change should be considered to increase people’s access to and continuation with physical activity interventions. However, technology-based interventions should be of interest to the target population. Evidence has suggested that an easy to use website providing step-by-step videos and interactive feedback is a facilitator to exercise among middle aged individuals [23]. These interventions could also be of benefit in providing professional support beyond the project, as people reported concerns over the lack of on-going support and it was seen as a barrier to continuing to exercise [14].

Financial costs and physical inaccessibility are well established barriers to physical activity [14, 23]. Providing free or subsidised exercise sessions incentivised the participation in the project. It has been suggested that targeting financial incentives to high-risk population may result in greater behaviour change success [24]. However, physical activity is a habitual behaviour and the use of financial incentives may assist in initiating the behaviour but may not necessarily result in long term behaviour change. Although it has been suggested that financial incentives are associated with longer-term smoking cessation [25], it is not clear whether changes in physical activity will be maintained. Lack of transportation and inconvenient timing of classes also contributed to the difficulty to access physical activity interventions [14, 23]. Previous evidence has also suggested that neighbourhood safety and weather conditions are key factors contributing to inaccessibility [14, 23]. Offering behaviour change programmes in multiple accessible locations could improve people’s engagement with physical activity. Furthermore, providing older adults and individuals with disabilities with appropriate transport may increase their engagement with the programme.

The coordination between primary care professionals and ERS provide a great opportunity for those wanting to change. Primary care presents an important platform to initiating health behaviour change due to its longitudinal nature. However, according to this study’s participants, GPs were not fully committed to referring eligible patients to the project. A recent qualitative study suggested that although primary care professionals believed in the importance of referring patients to external lifestyle services, several obstacles were acknowledged that were faced by patients and healthcare professionals [26]. These difficulties included long waiting lists, service discontinuation due to budget cuts, costs, keeping track of what is available and uncertainty of whether or not patients will take up the referral [26, 27]. Continued follow-up of referrals made and improving communications between general practices and referral services is needed.

Participants in ERS in this study have reported making changes to their physical activity levels and overall lifestyles regardless of the reported barriers. These changes have also influenced changes to their families’ lifestyles. Participants in this study experienced positive effects on their physical and mental health as a result of increased physical activity levels. Previous evidence has suggested that ERS have the potential not only to increase adherence to physical activity [28, 29], but may also enhance long-term sustainability [30].

This study provides evidence of the potential benefits of ERS in improving physical activity levels among inactive populations. Future research should examine the use of interventions that provides continuous support and follow-up, including technology-based interventions. This is to ensure adherence to physical activity in the long-term. ERS should provide staff with skills required to deal with people of different health conditions and physical abilities. Improving the links and communication between ERS staff and healthcare professionals, including referrers, is essential to improve engagement to the project and overall outcomes.

### Strengths and limitations

This study is among the first to present qualitative findings relating to participants’ and providers’ experience with an ERS project. A strength of the study is that it included 35 in-depth interviews from the three distinct groups in the project. This helped to provide a comprehensive picture of the project operations and the challenges and facilitators faced by those involved. Using the TDF may have led to study participants discussing factors that may not be otherwise discussed [18]. The TDF enables looking beyond individual factors and considers environmental and social influences on behaviour. Study codes were checked by a second researcher who was independent from the data collection process. Through a process of consensus, the two researchers reached agreement. This procedure improved the transparency and credibility of this study interpretation [31].

The findings of this study may not be completely transferable beyond the current context. Our findings may be specific to this project and its setup and may not apply to other ERSs particularly those aimed at individuals with certain conditions e.g. diabetes. Due to data confidentiality, the project manager contacted and subsequently recruited participants for the interviews. This may have introduced bias and limited the representativeness of the sample from the project general population. Project participants who were not able to participate in the study may have had different experiences. We would also have liked to recruit more people in the study that had dropped out of the study at different points and more men participants for the study to establish potential barriers to entry, engaging and continuation in the project. Face to face interviews can produce social desirability bias [32], especially when discussing health behaviour change and implementation behaviour. The interviewer, however, was external to the project and other related organisations, with no conflicting roles or affiliations, which is believed to help in accessing more private accounts. We acknowledge that collection of demographic data differed for staff and participants. For example, ethnicity and deprivation level were not collected for staff members. Finally, theoretical saturation was not known to have been reached due to a relatively small sample of project staff.

## Conclusion

Providing free or subsidised classes along with providing continuous follow-up and support appears to facilitate the engagement in physical activity among socioeconomically deprived populations. ERS needs to be provided in a friendly and non-judgemental environment to facilitate engagement in the scheme, as they have the potential not only to improve physical activity level but also provide an opportunity for social interaction and relationship building. Project participants required different healthcare needs, which calls for the need of skilled and qualified staff to work with various health conditions. There is a need to improve the awareness of available referral schemes and to enhance the communication between referring groups and scheme staff.

## Supplementary information

**Additional file 1.** Exploring stakeholder’s experiences’ of the ALFA project.

## Data Availability

The datasets generated and analyzed during the current study are not publicly available due to the inclusion of individual information that could compromise confidentiality. Requests for data sharing should be directed to Martin Gulliford: martin.gulliford@kcl.ac.uk .

## Abbreviations

ALFA: Active lifestyles for all
ERS: Exercise referral schemes
GPs: General practitioners
IPAQ: International Physical Activity Questionnaire
TDF: Theoretical domains framework
