# Supplementary material for: A community-based, sport-led programme to increase physical activity in an area of deprivation: a qualitative case study
Source: BMC Public Health. 2020 Jun 29;20:1018. doi: 10.1186/s12889-020-08661-1 (PMC7322853; doi:10.1186/s12889-020-08661-1)
Supplement: Supplementary file 1 — Additional file 1. Exploring stakeholder’s experiences’ of the ALFA project. [file 12889_2020_8661_MOESM1_ESM.pdf]

Exploring stakeholder's experiences' of the ALFA project

Interview schedule

Introduction

Thank you for agreeing to take part in our study. We are looking at people's experience of the '*Active Lifestyles for All*' project. This is a free physical activity programme in which 13 sessions are offered to local residents classed as inactive.

We are interested in what people in the project thought of it. I just want to ask you a few questions about your thoughts-please feel free to answer as honestly as possible. There are no right and wrong answers. All information will be kept completely confidential. Taking part in will not affect your involvement in the project at all. If you would prefer not to answer any of the questions that is fine and we can stop the interview at any point if you do not wish to continue. Are there any questions you would like to ask me before we start?

DEMOGRAPHICS?

May I turn on the recorder now?

Semi-structured interview schedule

For those who took part or are taking part in the project

So I'd just like you to tell me about your experiences of ALFA.

1. **How did you come to be on the ALFA programme?**

   1a. Where you referred by someone/did you self-refer?

   1b. Did you receive information about the project?

   1c. When did you start (and finish) the programme?

2. How was your initial meeting with a mentor?

   2a. What sort of things did you discuss?

   2b. Did you keep in touch with a mentor throughout the process?

3. **What were your initial thoughts and expectations of the programme?**

   3a. How did you think it would run?

   3b. How has it run?

4. How confident were you about taking part in the project and going to the classes?

5. Did you experience any difficulties attending the classes?

5a. In terms of time, cost, scheduling?

5a. How did you get there?

5c. Was there anything that would have made it easier for you to attend?

6. Do you think you will continue attending classes?

6a. How difficult/easy is it/has it been for you to continue with the classes or increase your physical activity?

6b. For instance, with other commitments?

6c. How easy was it to remember to attend?

7. What did you think about the classes offered?

8. Some people say they feel anxious or stressed about participating in group-based physical activity. Do you/have you experience/d anything like this in this project?

9. Did you know anyone that was taking part in the exercise classes because of the project?

9a. Did that have an impact on whether you joined or attended?

10. Were you prompted by someone (e.g. friend or family member; not necessarily involved in the project) to sign up to the project or attend the classes?

10a. Were you discouraged by anyone?

11. Before using this centre/ being in ALFA were there other sports facilities that you had access to?

11a. What were these?

12. Are there any other factors (that we haven't already discussed) that had an effect on you taking part?

13. Do you think you have gained skills from the project?

13a. If so, what were these?

13b. **How do you/have you approach(ed) your physical activity habits? Day to day?**

14. **Is there anything about the project that you think would put people off from becoming involved or from staying involved?**
    15a. What were the disadvantages of the project?

15. How do you feel now about the project?

16. **Was it a positive experience?**
    16a. How would you say you benefitted from participating in the project?
    16b. What do you think are the benefits of this programme?

17. **How confident are you that having taken part in this project will help for you to increase your physical activity in the future?**

18. Do you have any other comments or feedback about ALFA?

Thank you very much for your time and taking part in the interview today.

Any questions about the research.

Exploring stakeholder experiences of the ALFA project

Interview schedule

Introduction

Thank you for agreeing to take part in our study. We are looking at people's experience of the '*Active Lifestyles for All*' project. This is a free physical activity programme in which 13 sessions are offered to local residents classed as inactive.

We are interested in what people involved in the project thought of it. I just want to ask you a few questions about your thoughts-please feel free to answer as honestly as possible. There are no right and wrong answers. All information will be kept completely confidential. If you would prefer not to answer any of the questions that is fine and we can stop the interview at any point if you do not wish to continue. Are there any questions you would like to ask me before we start?

May I turn on the recorder now?

Semi-structured interview schedule

For those who were mentors in the project

1. **So I'd like to hear about your experience with ALFA. Could you tell me a bit about it?**

   1a. When did you become involved with ALFA?

   1b. Have you ever worked on a similar project?

2. What were your initial thoughts and expectations of the project?

   2a. How did you think it would run?

   2b. How has it run?

3. What would you say is your role/responsibilities/task/job within the project?

   3a. Is this your only role? If not what other roles/jobs/commitments do you have?

   3b. How does this fit in with your other commitments?

   3c. How easy is it for you to get here?

4. Do you feel like you were properly trained and briefed for your role?

   4a. Have you got sufficient resources to complete your role?

   4b. Was there support available when needed?

   4c. Is there anything about your role that you feel needed adapting?

   4d. What additional training if any, do you think would be useful?

5. Were you involved in the recruitment of any participants? E.g. at the drop-in clinics in GP's?

   5a. What did you think of the recruitment?

   5b. Did you refer people from other sections of your working life?

6. When people came along for their initial meeting what were their expectations and how much did they know about the project and what it involved?

7. **How do you/have you approach(ed) getting people to change their physical activity habits? Day to day?**

8. Have you had any issues engaging people with the programme/attending classes?

   8a. How comfortable have you been trying to overcome these issues?

   8b. Would you say that there are issues with a lack of confidence in participants?

9. Some people become stressed and anxious when starting to exercise after a long period of time or in group settings. Did anyone express any emotions like this to you?

10. **Has anyone expressed to you any difficulties attending the classes?**

    10a. In terms of time, cost, scheduling maybe? /How they got there?

    10b. Do you think this was a good location for the project? Any issues with it?

    10c. Was there anything that you think would have made it easier for people to attend?

    10d. Was there a system set up for when people do not attend classes?

11. Are there any other barriers we haven't discussed to people completing the whole 13-week block?

    11a. What about taking part in follow-ups?

    11b. What is the system for keeping track of people and following them up?

12. At follow-up sessions did you use feedback e.g. comparing one IPAQ with another from one session to the next e.g. 3months-6months?

    12a. Did it have an effect their behaviour?

13. Did you use evernote? Is there anything that we haven't discussed here that you may have recorded in evernotes? E.g. when people couldn't attend…

14. Did people know others in the class?

    13a. Did people start telling and bringing along their friends?

    13b. In your opinion did this have an impact on if people attended and stayed involved?

15. **What do you think the project gave participants in terms of skills?**

    14a. What do you think they thought about their ability to take part in classes?

    14b. How do you think the project affected people's goals of physical activity?

16. What is your opinion on whether the project will have an impact on people's physical activity in the future?

    15a. Have people said anything to you about this?

    15b. In terms of fitting it into their everyday lives? Setting targets and goals?

17. Do you think this is an suitable programme for everyone that is referred?

18. NEW for interview 4: What do you think about the range of classes offered to participants ?

19. Do **think it is a good idea/project?**

    17a. What are the benefits?

    17b. What has worked well?

    17c. Have you found it a worthwhile project to be involved in?

20. Do you any other comments of feedback about the project?

Thank you very much for your time and taking part in the interview today.

Any questions about the research.
